# Detection of Photofrin Fluorescence From Malignant and Premalignant Lesions in the Bronchus using a Full-color Endoscopic Fluorescence Imaging System

**DOI:** 10.1155/DTE.7.187

**Published:** 2001

**Authors:** Yoshinobu Ohsaki, Kaneyoshi Takeyama, Shoko Nakao, Sachie Tanno, Eri Toyoshima, Kyoko Nakanishi, Yutaka Nishigaki, Toshiyuki Ogasa, Shinobu Osanai, Kenjiro Kikuchi, Susumu Nakajima

**Affiliations:** 1 First Department of Medicine Asahikawa Medical College 2-1-1-1 Midorigaoka Higashi Asahikawa Hokkaido 078-8510 Japan; 2 Hamamatsu Photonics 812 Jokocho Hamamatsu Shizuoka 431-3196 Japan; 3 Obihiro University of Agriculture and Veterinary Medicine 2-11 Inadacho Nishi Obihiro Hokkaido 080-8555 Japan

## Abstract

*Study objectives*: To detect invisible lung cancer and to determine field of laser radiation during PDT we developed a full-color fluorescence fiberscopic system. We tested the efficacy of this system in patients with various bronchial malignancies.

*System design*: A fiber-optic endoscope was attached to a camera box containing a color ICCD camera which can detect from 400 to 700nm fluorescence in full-color. Light of average wavelength 405 nm was selected and radiated through the light channel of the fiberscope from a 300W Xenon lamp.

*Patients and methods*: We examined nine consecutive patients with bronchial malignancy admitted in our hospital to receive PDT. Sixteen lesions in these nine patients were observed with white light and excitation light and the results were compared. Histological examinations were done by taking biopsy specimens and samples for pathological and cytological examination. After the diagnosis was confirmed, 2.0 mg/kg Photofrin was injected. Forty eight hours after the administration of Photofrin, observation of the bronchial wall was made using a full-color endoscopic fluorescence imaging system just before PDT.

*Results*: Bright red fluorescence from Photofrin was Observed in 14/14 bronchial malignancies: 3 squamous cell carcinoma, 9 squamous cell carcinoma *in situ*, 1 metastatic breast cancer and 1 metastatic islet cell tumor. Bright red fluorescence was also detected in 2/2 squamous dysplasia. Green autofluorescence was observed in the normal part of the bronchus.

*Conclusions*: Results of the present study suggest that the full-color endoscopic fluorescence imaging system can be used to detect malignant and premalignant lesions as red fluorescence against green autofluorescence with Photofrin administration, and this system has the potential to detect absence of autofluorescence in cancerous lesions.


*Diagnostic and Therapeutic Endoscopy*, Vol. 7, pp. 187–195
Reprints available directly from the publisher
Photocopying permitted by license only

© 2001 OPA (Overseas Publishers Association) N.V.
Published by license under
the Harwood Academic Publishers imprint,
part of Gordon and Breach Publishing
a member of the Taylor & Francis Group.
All rights reserved.

### Detection of Photofrin Fluorescence from Malignant and Premalignant Lesions in the Bronchus using a Full-color Endoscopic Fluorescence Imaging System: A Preliminary Report

YOSHINOBU OHSAKI[a,*], KANEYOSHI TAKEYAMA[b], SHOKO NAKAO[a], SACHIE TANNO[a], ERI TOYOSHIMA[a], KYOKO NAKANISHI[a], YUTAKA NISHIGAKI[a], TOSHIYUKI OGASA[a], SHINOBU OSANAI[a], KENJIRO KIKUCHI[a] and SUSUMU NAKAJIMA[c]

[a]*First Department of Medicine, Asahikawa Medical College, 2-1-1-1 Midorigaoka Higashi, Asahikawa, Hokkaido 078-8510, Japan;* [b]*Hamamatsu Photonics, 812 Jokocho, Hamamatsu, Shizuoka 431-3196, Japan;* [c]*Obihiro University of Agriculture and Veterinary Medicine, 2-11 Inadacho Nishi, Obihiro, Hokkaido 080-8555, Japan*

*(Received 5 October 2001; Revised 26 November 2001; In final form 26 November 2001)*

*Study objectives*: To detect invisible lung cancer and to determine field of laser radiation during PDT we developed a full-color fluorescence fiberscopic system. We tested the efficacy of this system in patients with various bronchial malignancies.

*System design*: A fiber-optic endoscope was attached to a camera box containing a color ICCD camera which can detect from 400 to 700 nm fluorescence in full-color. Light of average wavelength 405 nm was selected and radiated through the light channel of the fiberscope from a 300 W Xenon lamp.

*Patients and methods*: We examined nine consecutive patients with bronchial malignancy admitted in our hospital to receive PDT. Sixteen lesions in these nine patients were observed with white light and excitation light and the results were compared. Histological examinations were done by taking biopsy specimens and samples for pathological and cytological examination. After the diagnosis was confirmed, 2.0 mg/kg Photofrin was injected. Forty eight hours after the administration of Photofrin, observation of the bronchial wall was made using a full-color endoscopic fluorescence imaging system just before PDT.

*Results*: Bright red fluorescence from Photofrin was observed in 14/14 bronchial malignancies: 3 squamous cell carcinoma, 9 squamous cell carcinoma *in situ*, 1 metastatic breast cancer and 1 metastatic islet cell tumor. Bright red fluorescence was also detected in 2/2 squamous dysplasia. Green autofluorescence was observed in the normal part of the bronchus.

*Conclusions*: Results of the present study suggest that the full-color endoscopic fluorescence imaging system can be used to detect malignant and premalignant lesions as red fluorescence against green autofluorescence with Photofrin administration, and this system

*Corresponding author. Tel.: +81-166-68-2442. Fax: +81-166-68-2449. E-mail: yohsaki@asahikawa-med.ac.jp has the potential to detect absence of autofluorescence in cancerous lesions.

*Keywords*: Cancer; Detection; Endoscope; Fiberscope; Hematoporphyrin derivatives; Photodynamic diagnosis

*Abbreviations*: LIFE, light-induced fluorescence endoscopy; PDT, Photodynamic therapy

### INTRODUCTION

PDT is a therapeutic modality used against cancers involving the use of a photosensitizer and the local application of laser [1,2]. A hematoporphyrin derivative, Photofrin (dihematoporphyrin ether and/or ester) (Axcan Pharma, Quebec, Canada) is used as the photosensitizer in PDT. Hematoporphyrin derivatives exhibit red fluorescence when light of wavelengths around 405 nm is radiated [3]. It is reported that Photofrin is retained selectively by tumor tissue compared with most normal tissues [4] except the liver, spleen, and kidney [5,6]. The fluorescence of hematoporphyrin derivatives *in vivo* has been reported in cancers of the lung [7], the bladder [8], and other sites [9]. Therefore, the difference in concentration of hematoporphyrin derivatives in malignant tissue versus that in surrounding normal tissue is the basis of fluorescence detection and photodynamic diagnosis.

Normal tissue exhibits green fluorescence [10] from collagen, nicotinamide-adenine dinucleotide phosphate and flavin-adenine dinucleotide [11,12] when excitation light is applied. Three different systems, LIFE lung system [13] (Xillix, Richmond, Canada), SAFE-1000 [14] (Asahi Optical, Tokyo, Japan) and D-Light AF system [15] (Storz, Tuttlingen, Germany), to detect absence of the autofluorescence in malignant lesions have been introduced. The LIFE lung system has two black and white CCDs while SAFE-1000 has one black and white CCD; these systems show black and green images. The D-Light AF system has a color CCD; it shows black and green images in the autofluorescence mode and dark-red and purple images in the aminolevulinic acid (ALA) mode. Images from these systems, even in the ALA mode of the D-Light AF system, were quite different from full-color fluorescence images.

ALA has been used for photodetection of early-stage lung carcinoma and malignant glioma [16–18]. ALA exhibits stronger red fluorescence than Photofrin by application of the excitation light, and the red fluorescence can be detected by the naked eye. However, in dark conditions, color information cannot be detected by unaided vision. Moreover, the sensitivity is not good enough to detect auto-fluorescence from the normal tissue. Development of detection techniques of fluorescence from cancerous tissue and normal tissue in full-color will define the location or field spread of cancer, thereby improving the planning of cancer treatment.

Such color fluorescence observation has not been possible because the sensitivity of previous CCDs and image intensifiers was not satisfactory. We recently developed a high-sensitive color intensified CCD (ICCD) endoscopic system which enables detection of 400–700 nm fluorescence in full-color. We believe that the introduction of this visual technology to medical practice will enable discovery of unexpected cancer lesions during PDT, improved definition of the field for laser radiation, and color spectrum analysis of the lesion.

We tested the feasibility of this system in patients with various bronchial malignancies. In this report, we present results of color fluorescence detection in nine cases with a variety of bronchial lesions using a high sensitive color ICCD endoscopic system.

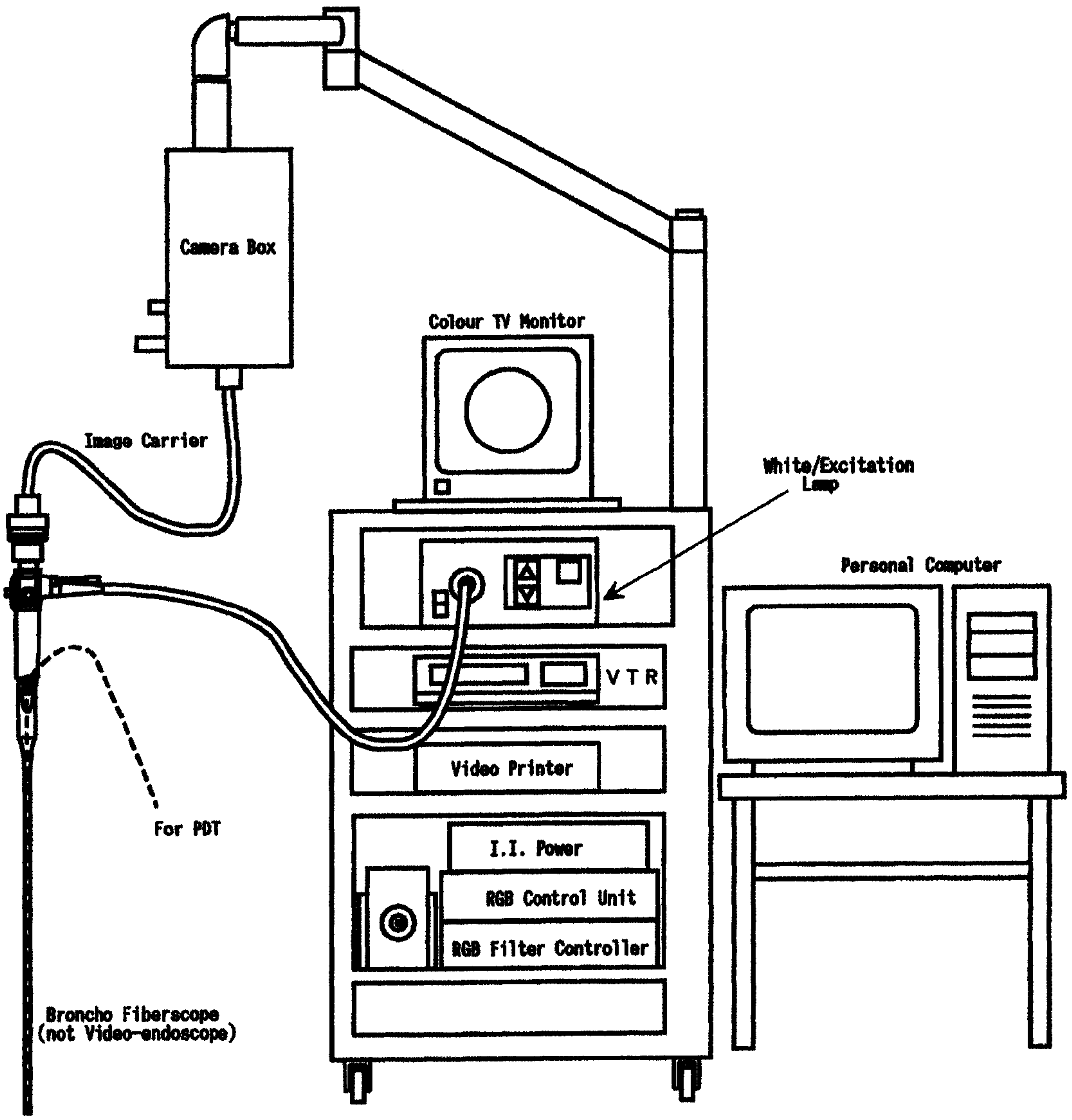

FIGURE 1 Block diagram of the high sensitive full-color fluorescence endoscopic system.

### MATERIALS AND METHODS

### Full-color Endoscopic Fluorescence Imaging System [19]

A fiber-optic endoscope was attached to a camera box containing a color ICCD camera (Hamamatsu Photonics, Hamamatsu, Japan). The camera box was connected to a RGB control unit with a RGB frame memory, image average system, scan converter and camera control unit. Light of averaged wavelength 405 nm was selected and radiated through the light channel of the fiberscope from a 300 W Xenon lamp. This wavelength excitation light was selectively attenuated in observations by a filter, which was designed for this system. White light from a 300 W Xenon lamp was used for white-light observation. Data from the color ICCD camera was input to the

TABLE I Results of fluorescence observation using the fluorescence imaging system

| Case | Sex | Age | Site | Histology | Stage | Red fluorescence† |
|---|---|---|---|---|---|---|
| 1 | M | 51 | L.M. | Islet | Meta. | Bright |
| 2 | F | 48 | R.TI | Adeno | Meta. | Bright |
| 3 | M | 74 | $R.B_9$ | SqCC | Tis* | Bright |
| 4 | M | 73 | $L.B_6$ | SqCC | Ia | Bright |
| 5 | M | 65 | $L.B_3$ | SqCC | Ia | Bright |
| 6 | M | 56 | L.Lo | SqCC | Ia* | Bright |
| 7 | M | 81 | L.U. | SqCC | Tis* | Bright |
| | | | L.U. | Normal | | Weak |
| | | | R.U. | Normal | | Weak |
| 8 | M | 70 | $L.B_4$ | SqCC | Tis | Bright |
| | | | $L.B_{1+2}$ | Dysplasia | | Bright |
| | | | $R.B_3$ | Dysplasia | | Bright |
| 9 | M | 76 | $R.B_1$ | SqCC | Tis | Bright |
| | | | $R.B_2$ | SqCC | Tis | Bright |
| | | | R.S. | SqCC | Tis | Bright |
| | | | $L.B_3$ | SqCC | Tis | Bright |
| | | | L.Us/Ls | SqCC | Tis | Bright |
| | | | L.Us | SqCC | Tis | Bright |

L, left; R, right; M, main bronchus; TI, truncus intermedius; Lo, lower lobe; U, upper lobe; R.S., right seconcarina; Us, upper segment; Ls, lingular segment; Us/Ls, bifurcation of Us/Ls; Islet, islet cell tumor; Adeno adenocarcinoma; SqCC, squamous cell carcinoma; Meta., metastasis.
* Case with second primary.
† Cases in which red fluorescence was detected.

Olympus TV system, OTV-F3 (Olympus, Tokyo, Japan). There was a selector to switch between fluorescence observation and white light observation. Output devices were a color monitor, a Macintosh computer and a video recorder (Fig. 1). Any kind of endoscope with OES attachment can be used because the system has an Olympus Endoscopy System (OES) attachment.

### Photosensitizer and PDT

Photofrin was obtained from Wyeth-Lederle Japan (Tokyo, Japan) at a concentration of 2.5 mg/ml in sterile saline. Photofrin was injected into the vein at a dose of 2.0 mg/kg 48 h prior to PDT. This was an approved concentration for PDT. After the observation using the full-color endoscopic fluorescence imaging system, patients received PDT at the same lesion. PDT was done by 75–200 J/cm$^2$ excimer dye laser radiation using EDL-PDT1 (Hamamatsu Photonics, Hamamatsu, Japan).

### Patients

Nine consecutive patients with bronchial malignancy admitted our hospital to receive PDT from May 2000 to July 2001 entered this study. Sixteen lesions in these nine patients were observed with white light and with excitation light and its results were compared. Three patients had squamous cell carcinoma of the bronchus, four had squamous cell carcinoma *in situ*, one had pulmonary metastatic islet cell tumor from the pancreas and one had bronchial metastasis from breast cancer. One patient with carcinoma *in situ* had two different lesions with squamous dysplasia. Pathological diagnoses of the lesions had been confirmed before admission. It is reported that biopsy sites uptake porphyrin derivatives thereafter exhibit red fluorescence. To avoid this artifact, these patients underwent photodynamic diagnosis and PDT more than two weeks after the biopsies. Table I shows characteristics of the patients.

### Endoscopic Observations

Observations of the bronchus were made by a conventional bronchoscopy using white light in all cases to confirm pathological diagnosis and location

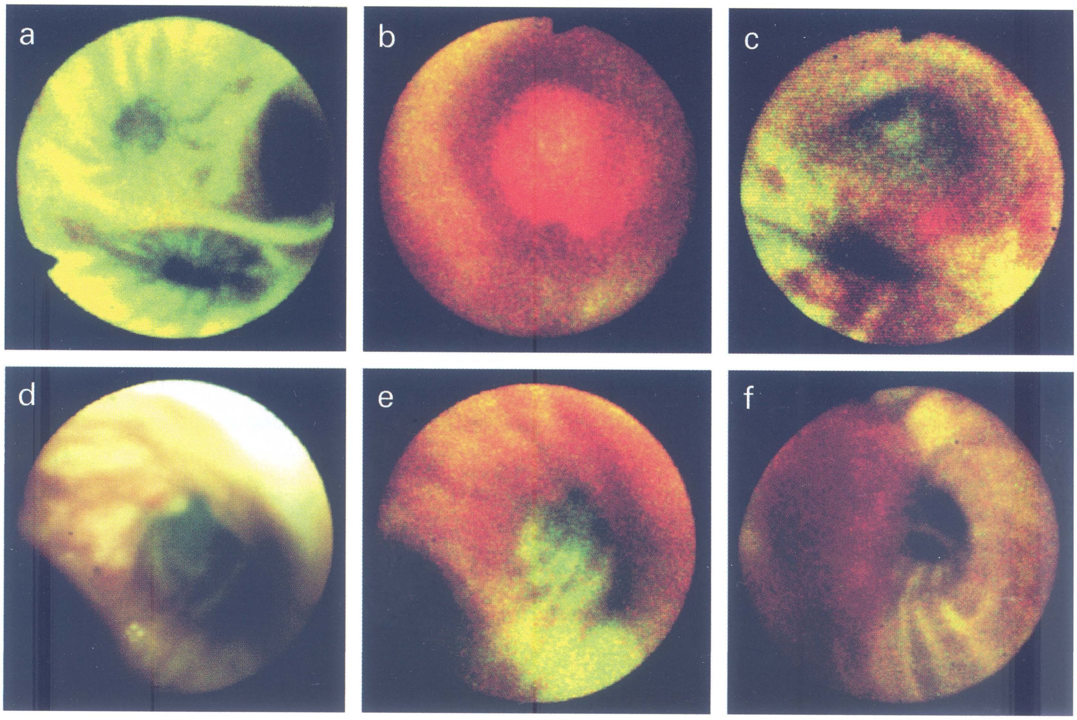

FIGURE 2 Full color fluorescence images of the bronchus. Green autofluorescence was observed from normal bronchial wall (a). Red bright fluorescence was detected in metastatic breast tumor (b, case 2), in squamous cell carcinoma (c, case 4). In case 6, white-light observation showed granular change in upper part of the left $B_6$ (d) and red fluorescence was detected in the fluorescence mode (e). (f) shows fluorescence image in case 7 with carcinoma *in situ.*

of the tumor before Photofrin administration. Histological examinations were done by taking biopsy specimens and samples for pathological and cytological examination. After the diagnosis was confirmed, Photofrin was injected. Forty eight hours after the administration of Photofrin, observations of the bronchial wall were made using a full-color endoscopic fluorescence imaging system just before PDT.

All major branches of the bronchus were observed using the full-color endoscopic fluorescence imaging system. Color fluorescence images were compared to the white light images in the diagnosed lesions. Lesions were considered to be present when bright red fluorescence was detected in the same part of the bronchus. Shapes were compared between white light images and fluorescence images. Biopsy was considered when lesion with red fluorescence other than the diagnosed lesion was found.

Patients were well informed about the purpose and method of the fluorescence observation. All patients agreed to the observations using the new system in addition to the normal bronchofiberscopic observation. No extra premedication or anesthesia was given other than common medication for bronchofiberscopy and PDT. Olympus fiber-optic bronchoscopy, BF-type 40, was used in the fluorescence observations.

### RESULTS

Bright red fluorescence from Photofrin was observed in 14/14 bronchial malignancies: three squamous cell carcinoma, nine squamous cell carcinoma *in situ*, one metastatic breast cancer and one metastatic islet cell tumor (Table I). Bright red fluorescence was also detected in 2/2 squamous dysplasia. Yellow-green

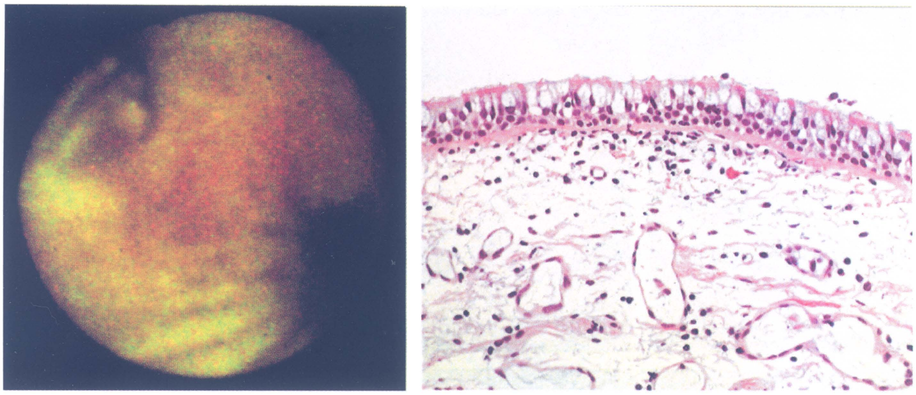

FIGURE 3 Left figure shows the weak red fluorescence which was observed in two different parts in case 7. No pathological abnormality was found (right).

fluorescence was observed in the normal bronchus in all cases with or without Photofrin injection (Fig. 2a). Red fluorescence was not detected in bronchial parts other than the lesion for PDT, although weak red fluorescence was observed in two different bronchial portions in case 7.

Figure 2b shows the fluorescence observation in case 2 with metastatic bronchial tumor from breast cancer; right trunchus intermedius was nearly obstructed with this tumor. Bright red fluorescence was observed in the fluorescence mode. The bronchial wall seemed to be red due to the reflection of red Photofrin fluorescence. Figure 2c shows fluorescence image of bifurcation of the left upper and lower bronchus in case 4 with squamous cell carcinoma. In the white-light observation, white thickness of the membrane was noticed on the bifurcation. The lesion exhibited red fluorescence from Photofrin. Figure 2d shows white-light observation in case 6. White thick lesion with granular change was found in the upper wall of the left $B_6$. In the fluorescence observation, bright red fluorescence was detected (Fig. 2e) as well as yellow-green fluorescence in the lower part. Figure 2f shows the fluorescence image in case 7. Thick lesion in the left upper lobe bronchus exhibited bright red fluorescence. In this case two different parts with weak red fluorescence were observed. No abnormality was found in the biopsy specimens from these lesions (Fig. 3). Other than these two normal bronchial parts

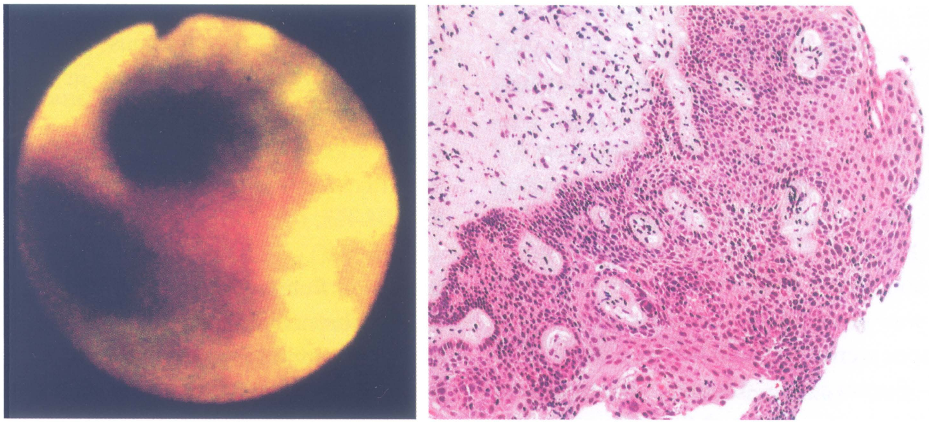

FIGURE 4 Fluorescence finding and pathological finding of squamous dysplasia in case 8.

with weak red Fluorescence in case 8, we did not find undiscovered cancer lesions in the present study.

Bright red fluorescence was observed in case 8 with carcinoma *in situ* in the left $B_4$. This case had two different lesions with squamous dysplasia. These lesions also exhibited bright red fluorescence. Figure 4 shows fluorescence observation and pathological finding of the lesion with squamous dysplasia.

### DISCUSSION

Lung cancer is the most common cause of cancer death in Japan and North America [20]. The overall five-year survival of patients with lung cancer is approximately 14%. In patients with early lung cancer, the five-year survival ranges from 40 to 85%. Most bronchogenic cancers are smoking related which tend to develop at multiple sites in the bronchus [21]. In fact, we observed five cases with multiple lesions including dysplasia in seven cases with bronchogenic squamous cell carcinoma in the present study. Case 9 had six different lesions with squamous cell carcinoma *in situ*. Carcinoma *in situ* is bronchoscopically visible in less than 30% of cases and microinvasive tumors are visible in only about two thirds of cases [22]. Therefore, detection of invisible cancer has long been a challenge. PDT is an effective method to treat early bronchial cancer. Clinical application of PDT is increasing because it is the most appropriate for treatment of early lung cancer for patients with impaired heart and lung function. Observation of fluorescence from Photofrin in cases of PDT will help to detect invisible early cancer and to determine the field for laser radiation.

Detection of invisible cancer using bronchoscopy has been tried in two different methods; detection of autofluorescence and detection of fluorescence from porphyrin derivatives. Lack of green autofluorescence in caner lesions can be detected using LIFE system [23] and SAFE-1000 system [24,14]. In the LIFE system, normal tissue is radiated with a blue laser beam to exhibit green fluorescence while the area with malignancy does not exhibit; the cancerous part is thereby recognized as a negative image of the green fluorescence. Clinical application of these systems significantly improved detection rate of invisible cancer of the bronchus [22,25]. The LIFE system was found to be 50% or more sensitive than white-light bronchoscopy in the detection of moderate/severe dysplasia and carcinoma *in situ* [26]. In contrast, the LIFE system did not improve detection of squamous metaplasia or dysplasia in current or former smokers [27]. Detection of red fluorescence of porphyrin derivatives in the cancer lesion has been tried [28]. Lam *et al.* reported detection of red fluorescence from Photofrin measuring the red and green ratio in early lung cancer using a low dose Photofrin injection; they detected four invisible early lung cancers in four patients using a ratio fluorometer probe [29]. D-Light AF system is designed to detect green autofluorescence as well as red fluorescence from ALA [15]. Although this system has the potential to detect fluorescence of other porphyrin derivatives in the ALA mode, simultaneous detection of different color fluorescence is not possible.

Our system made possible observation from 400 to 700 nm fluorescence in full-color, although the averaged 405 nm was attenuated because this is the wavelength of excitation light. The source of fluorescence in this particular lesion can be determined by observing color image of the portion; for example, green fluorescence was observed from the normal tissue, red fluorescence from cancerous part and brown fluorescence from bleeding in case 4. In the present study, 16 out of 16 malignant and premalignant lesions were detected as lesions exhibiting red fluorescence with Photofrin administration. Difference in the red fluorescence between squamous cell carcinoma, carcinoma *in situ* and dysplasia was not distinguishable. Red fluorescence was not found in the bronchus other than in the prediagnosed cancerous or dysplastic lesions in the present study, except for the weak reddishness observed in two different parts in case 7; no malignant changes were found in the biopsy specimens from these portions. The red fluorescence in these lesions was easily distinguishable from that of malignant lesions.

ALA induced protoporphyrin IX exhibits 630 nm fluorescence which is identical to the wavelength of

Photofrin fluorescence. The second generation hematoporphyrin derivatives are under development and exhibit fluorescence between 650 and 700 nm. Our system can detect fluorescence at these wavelengths and can, therefore, be used in the photodynamic diagnosis using porphyrin derivatives other than Photofrin and ALA.

Although a larger study population is needed to assess the advantage of full-color autofluorescence observation to detect early lung cancer in clinical practice, absence of autofluorescence in cancerous lesions was observed even without Photofrin administration in all cases in the present study. The results of the present study suggest that the full-color endoscopic fluorescence imaging system can be used to detect malignant and premalignant lesions; as red fluorescence against green autofluorescence with Photofrin administration, and this system has the potential to detect absence of autofluorescence in cancerous lesion. We believe that detection and diagnosis of malignant and premalignant lesions by analyzing color fluorescence images will be possible in the near future using a flourofiberscopy like ours.

### Acknowledgements

The authors thank Drs Kiyoko Shibukawa, Takaaki Sasaki, Yoko Aburakawa, Junko Chinda, Yasushi Yamamoto, Hiroshi Ide, and Hitoshi Nakano for their help in this study. Yoshinobu Ohsaki is grateful to Simon N. Bayley for the English revision.

### References

[1] Kato, H., Horai, T., Furuse, K., Fukuoka, M., Suzuki, S., Hiki, Y., Ito, Y., Miura, S., Tenjin, Y., Hisazumi, H. and Hayata, Y. (1993) "Photodynamic therapy for cancers: A clinical trial of porfimer sodium in Japan", *Jpn J. Cancer Res.* **84**, 1209–1214.

[2] Dougherty, T.J., Gomer, C.J., Henderson, B.W., Jori, G., Kessel, D., Korbelik, M., Moan, J. and Peng, Q. (1998) "Photodynamic therapy", *J. Natl Cancer Inst.* **90**, 889–905.

[3] Eales, L. (1978) "Clinical chemistry of the porphyrins", In: Dolphyin, D., ed, The Porphyrins (Academic Press, New York), p 810.

[4] Nakajima, S., Hayashi, H., Omote, Y., Yamazaki, Y., Hirata, S., Maeda, T., Kubo, Y., Takemura, T., Kakiuchi, Y., Shindo, Y., *et al.*, (1990) "The tumour-localizing properties of porphyrin derivatives", *J. Photochem. Photobiol. B* **7**, 189–198.

[5] Gomer, C.J. and Doughty, T.J. (1979) "Detection of [$^3$H]- and [$^{14}$C] hematoporphyrin derivative distribution in malignant and normal tissue", *Cancer Res.* **39**, 146–151.

[6] Bellnier, D.A., Ho, Y.K., Pandey, R.K., Missert, J.R. and Dougherty, T.J. (1989) "Distribution and elimination of Photofrin® II in mice", *Photochem. Photobiol.* **50**, 221–228.

[7] Hayata, Y., Kato, H., Ono, J., Matsushima, Y., Hayashi, N., Saito, T. and Kawate, N. (1982) "Fluorescence fiberoptic bronchoscopy in the diagnosis of early stage lung cancer", *Recent Results Cancer Res.* **82**, 121–130.

[8] Benson, R.C., Farrow, G.M., Kinsey, J.H., Cortese, D.A., Zincke, H. and Utz, D.C. (1982) "Detection and localization of *in situ* carcinoma of the bladder with hematoporphyrin derivative", *Mayo Clin. Proc.* **57**, 548–555.

[9] Lipson, R.C., Baldes, E.J. and Olsen, A.M. (1961) "The use of a derivative of hematoporphyrin in tumor detection", *J. Natl Cancer Inst.* **26**, 1–11.

[10] Schomacker, K.T., Frisoli, J.K., Compton, C.C., Flotte, T.J., Richter, J.M., Deutsch, T.F. and Nishioka, N.S. (1992) "Ultraviolet laser-induced fluorescence of colonic polyps", *Gastroenterology* **102**, 1155–1160.

[11] Lakowicz, J.R. (1983) Principles of fluorescence spectroscopy (Plenum Press, New York).

[12] Schomacker, K.T., Frisoli, J.K., Compton, C.C., Flotte, T.J., Richter, J.M., Nishioka, N.S. and Deutsch, T.F. (1992) "Ultraviolet laser-induced fluorescence of colonic tissue: basic biology and diagnostic potential", *Lasers Surg. Med.* **12**, 63–78.

[13] Palcic, B., Lam, S., Hung, J. and MacAulay, C. (1991) "Detection and localization of early lung cancer by imaging techniques", *Chest* **99**, 742–743.

[14] Kakihana, M., Il, K.K., Okunaka, T., Furukawa, K., Hirano, T., Konaka, C., Kato, H. and Ebihara, Y. (1999) "Early detection of bronchial lesions using system of autofluorescence endoscopy (SAFE) 1000", *Diagn. Ther. Endosc.* **5**, 99–104.

[15] Leonhard, M. (1999) "New incoherent autofluorescence/fluorescence system for early detection of lung cancer", *Diagn. Ther. Endosc.* **5**, 71–75.

[16] Kriegmair, M., Zaak, D., Stepp, H., Baumgartner, R., Knuechel, R. and Hofstetter, A. (1999) "Transurethral resection and surveillance of bladder cancer supported by 5-aminolevulinic acid-induced fluorescence endoscopy", *Eur. Urol.* **36**, 386–392.

[17] Leunig, A., Betz, C.S., Mehlmann, M., Stepp, H., Arbogast, S., Grevers, G. and Baumgartner, R. (2000) "Detection of squamous cell carcinoma of the oral cavity by imaging 5-aminolevulinic acid-induced protoporphyrin IX fluorescence", *Laryngoscope* **110**, 78–83.

[18] Peng, Q., Warloe, T., Berg, K., Moan, J., Kongshaug, M., Giercksky, K.E. and Nesland, J.M. (1997) "5-Aminolevulinic acid-based photodynamic therapy. Clinical research and future challenges", *Cancer* **79**, 2282–2308.

[19] Ohsaki, Y., Nishigaki, Y., Takeyama, K., Nakanishi, K., Ide, H., Matsumoto, K., Saito, H., Osanai, S., Kikuchi, K. and Nakajima, S. (2000) "Visualization of cancer using high sensitive fluorodynamiccamera and fiber-optic endoscope", *Porphyrins* **9**, 197–203.

[20] Omenn, G.S. (2000) "Cancer prevention", In: Goldman, L. and Bennett, J.C., eds, Textbook of Medicine (W.B. Saunders, Philadelphia), pp 1032–1035.

[21] Miller, Y.E. (2000) "Pulmonary neoplasms", In: Goldman, L. and Bennett, J.C., eds, Textbook of Medicine (W.B. Saunders, Philadelphia), pp 449–455.

[22] Lam, S., Kennedy, T., Unger, M., Miller, Y.E., Gelmont, D., Rusch, V., Gipe, B., Howard, D., LeRiche, J.C., Coldman, A. and Gazdar, A.F. (1998) "Localization of bronchial intraepithelial neoplastic lesions by fluorescence bronchoscopy", *Chest* **113**, 696–702.

[23] George, P.J. (1999) "Fluorescence bronchoscopy for the early detection of lung cancer", *Thorax* **54**, 180–183.

[24] Adachi, R., Utsui, T. and Furusawa, K. (1999) "Development of the autofluorescence endoscope imaging system", *Diagn. Ther. Endosc.* **5**, 65–70.

[25] Venmans, B.J., van der Linden, H., van Boxem, T.J., Postmus, P.E., Smit, E.F. and Sutedja, T.G. (1998) "Early detection of preinvasive lesions in high-risk patients", *J. Bronchol.* **5**, 280–283.

[26] Lam, S., MacAulay, C., Hung, C., LeRiche, J., Profio, A.E. and Pacic, B. (1993) "Detection of dysplasia and carcinoma *in situ* with a lung imaging fluorescence endoscope device", *J. Thorac. Cardiovasc. Surg.* **105**, 1035–1040.

[27] Kurie, J.M., Lee, J.S., Morice, R.C., Walsh, G.L., Khuri, F.R., Broxon, A., Ro, J.Y., Franklin, W.A., Yu, R. and Hong, W.K. (1998) "Autofluorescence bronchoscopy in the detection of squamous metaplasia and dysplasia in current and former smokers", *J. Natl Cancer Inst.* **90**, 991–995.

[28] Kato, H. and Cortese, D.A. (1985) "Early detection of lung cancer by means of hematoporphyrin derivative fluorescence and laser photoradiation", *Clin. Chest Med.* **6**, 237–253.

[29] Lam, S., Palcic, B., McLean, D., Hung, J., Korbelik, M. and Profio, E. (1990) "Detection of early lung cancer using low dose photofrin II", *Chest* **97**, 333–337.

